# Complete mitochondrial genomes of living and extinct pigeons revise the timing of the columbiform radiation

**DOI:** 10.1186/s12862-016-0800-3

**Published:** 2016-10-26

**Authors:** André E. R. Soares, Ben J. Novak, James Haile, Tim H. Heupink, Jon Fjeldså, M. Thomas P. Gilbert, Hendrik Poinar, George M. Church, Beth Shapiro

**Affiliations:** 1 Department of Ecology and Evolutionary Biology, University of California, Santa Cruz, 1156 High Street, Santa Cruz, CA 95064 USA; 2 Revive & Restore, The Long Now Foundation, San Francisco, CA 94123 USA; 3 Natural History Museum of Denmark, University of Copenhagen, Øster Voldgade 5-7, 1350 Copenhagen, Denmark; 4 Environmental Futures Research Institute, Griffith University, 170 Kessels Road QLD 4111, Nathan, Australia; 5 McMaster Ancient DNA Centre, Departments of Anthropology and Biology, and the Michael G. DeGroote Institute for Infectious Disease Research, McMaster University, 1280 Main Street West, Hamilton, ON L8S 4 L9 Canada; 6 Wyss Institute for Biologically Inspired Engineering, Harvard University, 3 Blackfan Circle, Boston, MA 02115 USA; 7 Department of Genetics, Harvard Medical School, 77 Avenue Louis Pasteur, Boston, MA 02115 USA

**Keywords:** Columbidae, Ancient DNA, time calibrated phylogeny, *Pezophaps solitaria*, *Raphus cucullatus*, Passenger pigeon

## Abstract

**Background:**

Pigeons and doves (Columbiformes) are one of the oldest and most diverse extant lineages of birds. However, the nature and timing of the group’s evolutionary radiation remains poorly resolved, despite recent advances in DNA sequencing and assembly and the growing database of pigeon mitochondrial genomes. One challenge has been to generate comparative data from the large number of extinct pigeon lineages, some of which are morphologically unique and therefore difficult to place in a phylogenetic context.

**Results:**

We used ancient DNA and next generation sequencing approaches to assemble complete mitochondrial genomes for eleven pigeons, including the extinct Ryukyu wood pigeon (*Columba jouyi*), the thick-billed ground dove (*Alopecoenas salamonis*), the spotted green pigeon (*Caloenas maculata*), the Rodrigues solitaire (*Pezophaps solitaria*), and the dodo (*Raphus cucullatus*). We used a Bayesian approach to infer the evolutionary relationships among 24 species of living and extinct pigeons and doves.

**Conclusions:**

Our analyses indicate that the earliest radiation of the Columbidae crown group most likely occurred during the Oligocene, with continued divergence of major clades into the Miocene, suggesting that diversification within the Columbidae occurred more recently than has been reported previously.

**Electronic supplementary material:**

The online version of this article (doi:10.1186/s12862-016-0800-3) contains supplementary material, which is available to authorized users.

## Background

The lineage of pigeons and doves, Columbiformes, is one of the most diverse non-passerine orders of birds. Columbiformes are the sixth most speciose order among the 40 traditionally recognized orders of living birds, according to species counts by the International Ornithologist’s Committee World Birdlist [1]. Pigeons and doves inhabit every land area outside the Arctic and Antarctic, and display a wide range of variation in their ecological adaptations, although their relatively conserved anatomy and morphology has obscured phylogenetic relationships within the family. Recent whole genome analyses resolved the placement of pigeons and doves as sister to sandgrouses (Pterocliformes) and mesites (Mesitornithiformes) [2, 3].

Previous genetic analyses have helped to clarify cryptic relationships among some branches of the Columbiformes, and have provided insights into the timing of the diversification of this group [4–11]. For example, ancient DNA extracted from the remains of two large flightless pigeons, the extinct dodo (*Raphus cucullatus*) and its sister species, the solitaire (*Pezophaps solitaria*), suggested that the closest living relative of these species is the Nicobar pigeon (*Caloenas nicobarica*) [12]. This work also suggested that the dodo and solitaire lineages diverged 18–36 Million years ago (Mya), during the late Oligocene [13]. This date was biogeographically interesting because it was prior to the emergence of the two islands to which the flightless species were endemic [12]. Similarly, old divergence estimates were obtained in a later study by Pereira et al. [5], who used a more taxonomically comprehensive phylogeny of both mitochondrial and nuclear DNA to infer that the dodo and solitaire diverged from the *Caloenas* lineage 33.5–50 Mya and from each other 15–30 Mya. This study also concluded that the entire columbiform lineage probably originated during the Cretaceous, and that the main period of diversification among columbiformes occurred at the Paleocene/Eocene boundary [5].

Here, we revisit the timing of the origin of and diversification within the columbiformes using a data set of complete mitochondrial genomes from a taxonomically broad selection of pigeons and doves. We assemble complete mitochondrial genomes from eleven pigeon species and from the yellow-throated sandgrouse, *Pterocles gutturalis*. Our new genomes include those of the extinct Ryukyu wood pigeon (*Columba jouyi*), the extremely rare thick-billed ground dove (*Alopecoenas salamonis*), which is known from only two specimens [14, 15], the spotted green pigeon (*Caloenas maculata*), which is also known as Liverpool pigeon and is represented by only one surviving museum specimen [16], the Rodrigues solitaire (*Pezophaps solitaria*), and the dodo (*Raphus cucullatus*). Using these newly assembled mitochondrial genomes and available published mitochondrial genomes, we estimate a phylogeny to infer the major evolutionary relationships among the pigeons and doves using both a Bayesian and maximum likelihood approach. Further, we use a molecular clock approach, calibrated using whole-genome data [2, 3], to infer the timing of divergence between Columbiformes, Pterocliformes, and the Galloansera.

## Results and discussion

### A new phylogeny for pigeons and doves

**Fig. 1:**
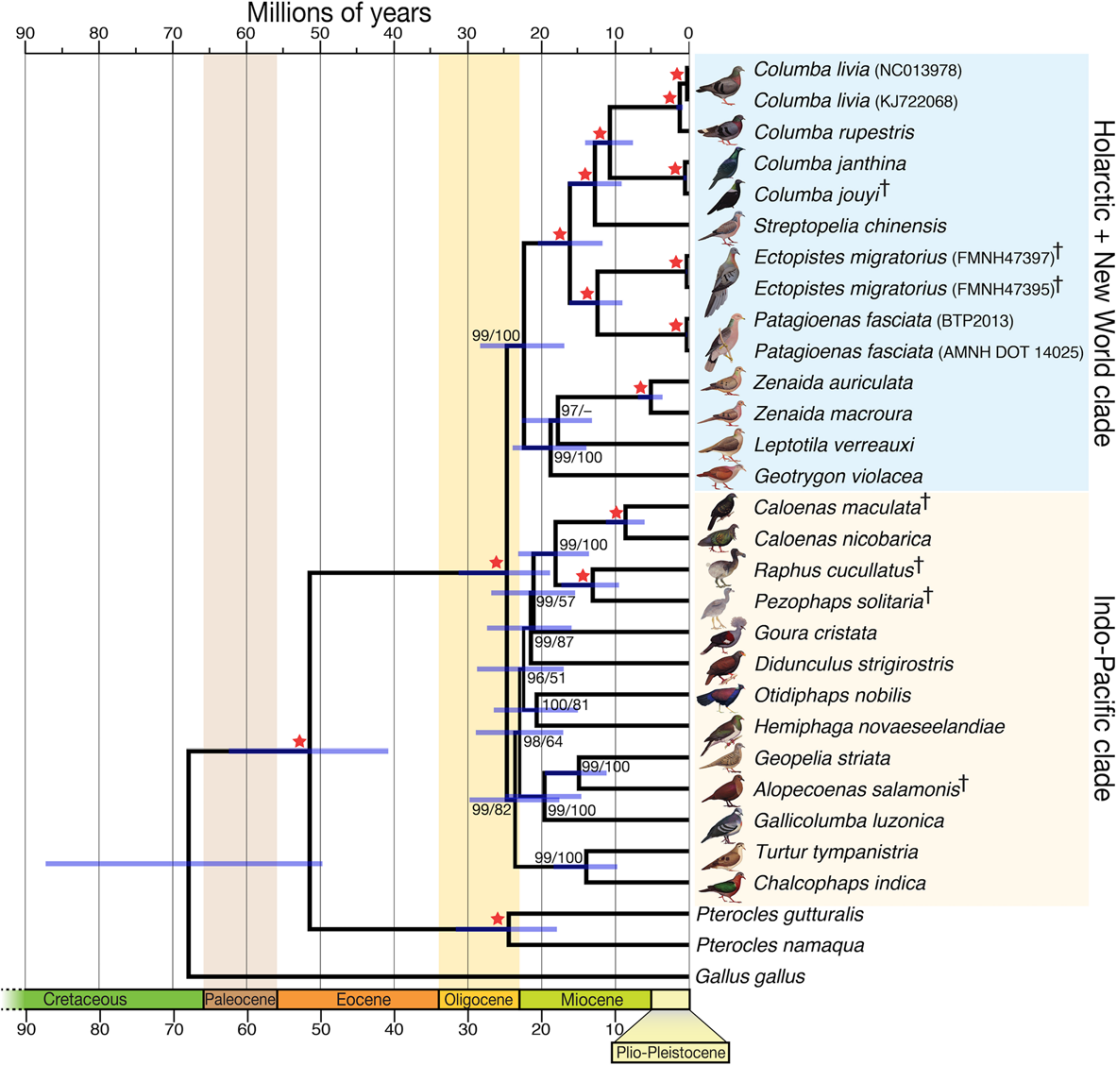
A molecular clock phylogeny for the pigeons and doves (Columbiformes). Star represents both 100 % Bayesian posterior probability and 100 % ML bootstrap support. En dash (−) indicates ML bootstrap values smaller than 50 %. Bars represent the 95 % CI for node ages, and † denotes extinct species. All, but *Raphus cucullatus* and *Pezophaps solitaria*, images reproduced from the book “Pigeons and Doves” by David Gibbs, Eustace Barnes and John Cox, reproduced with permission of the publishers, Bloomsbury Publishing

Both the ML and Bayesian approaches to inferring a mitochondrial phylogeny result in the same overall topology, with strong statistical support for most nodes (Fig. 1). The phylogeny supports two major clades, an Indo-Pacific clade (Fig. 1, *yellow*), and a Holarctic clade (Fig. 1, *blue*) that also includes New World pigeons. This or similar structure has been observed previously in more taxonomically focused data sets [5, 17, 18].

Within the Indo-Pacific clade, our results corroborate several previously supported relationships. Similarly to Jønsson et al. [4] and Moyle et al. [9], we find that *Alopecoenas* is more closely related to the Zebra dove (*Geopelia striata*) than it is to the Luzon bleeding-heart dove (*Gallicolumba luzonica*). Our results also support the previously identified close relationship between the dodo (*Raphus cucullatus*), solitaire (*Pezophaps solitaria*), and Nicobar pigeon (*Caloenas nicobarica*) [13], and the sister relationship between the Nicobar pigeon and the spotted green pigeon (*Caloenas maculata*) [16]. We also find that this group is sister to the crowned pigeons *Goura*, supporting the result of Shapiro et al. [13], but in contrast to Pereira et al. [5], who placed the *Caloenas*/*Raphus* lineage within the Didunculinae clade.

Also within the Indo-Pacific clade, our results suggest that the genus *Otidiphaps*, instead of belonging to the Didunculinae clade, is sister to the highly diverse fruit pigeon clade [8], which is represented in our phylogeny by *Hemiphaga*. Further investigation that includes other species that have been suggested to be closely related to these genera, such as the thick-billed ground pigeon (*Trugon terrestris*) [5], will help to further disentangle these evolutionary relationships.

Within the Holarctic and New World clade, we identify a strongly supported subclade that includes the genera *Leptotila*, *Zenaida* and *Geotrygon*. These species occur from North to South America and diverged from each other during the early Miocene. The close relationship between *Zenaida* and *Leptotila* conflicts with previous results [19] that placed the white-tipped dove, *Leptotila verreauxi*, closer to the violaceous quail-dove, *Geotrygon violacea*, than to *Zenaida*. The difference between our and previous phylogenies may be attributable to including the mourning dove, *Zenaida macroura*, in our analysis. The *Leptotila*/*Zenaida*/*Geotrygon* clade has strong support in the Bayesian analysis, but weak support in the Maximum Likelihood tree, highlighting the challenge of inferring and interpreting phylogenetic relationships from taxonomically limited data sets.

### Inferring the timing of diversification within pigeons and doves

The combination of recent genome-scale analyses of avian evolution [2] and our new data set of complete mitochondrial genomes provides an opportunity to recalibrate the timing of the origin of and radiation within the Columbiformes. When inferring time-calibrated phylogenies, careful consideration is required with respect to each fossil or type of calibration employed [20]. Theoretical and empirical work have shown that using multiple calibration points generally leads to more robust estimates of evolutionary rates [21, 22]. Unfortunately, no fossils are known from within the family of pigeons and doves that could be used as calibration [23, 24]. Therefore, we used the time-scales estimated by Jarvis et al. [2] and Prum et al. [3], which agree with each other with respect to the timing of diversification of Columbiformes.

We find that pigeons and doves most likely began to diversify during the late Oligocene, and continued to diversify into the Miocene (Fig. 1). Specifically, we find that the Holarctic and Indo-Pacific clades diverge around 24.7 Mya (95 % credibility interval, CI, 18.9–31.3 Mya), similar to [25], which place the divergence of two columbiform clades during the mid-Oligocene. This timing is in contrast to previous studies, which suggested that the Columbiformes radiated much earlier and more slowly, over the course of the Eocene and Oligocene [5]. The transition from the Eocene to the Oligocene corresponds to a period of widespread global cooling [26, 27] and associated geological changes, including the opening of the Drake Passage and the formation of the Wallacea region [28]. These changing global conditions may in part explain the timing of the rapid diversification within the Columbidae, which contains many highly dispersive, “supertramp” species [29].

We estimate that the dodo and solitaire diverged around 13.1 Mya (95 % CI 9.5–17.3 Mya), during the Early/Middle Miocene transition, rather than around the Oligocene/Miocene transition (22.8 Mya [5] and 25.6 Mya [13]), as previously proposed. We also find that the common ancestor of the dodo and solitaire diverged from *Caloenas* around 18 Mya (95 % CI 13.6–23.2 Mya), rather than during the Middle or latest Eocene (33.6 Mya [5] and 42.6 Mya [13]). Although our estimated divergence dates are more recent than those proposed previously, these dates indicate that both flightless pigeons diverged from their closest flying relative at least 10 Mya prior to the emergence of Mauritius and Rodrigues Islands, to which the flightless birds were endemic [12, 30, 31]. This finding corroborates previous claims that these lineages must have originated elsewhere [13]. The passenger pigeon, *Ectopistes migratorius,* is known to be closely related to the lineage of large New Word pigeons, represented in our study by the band-tailed pigeon, *Patagioenas fasciata* [10, 11]. However, the timing of divergence between these lineages has been unknown. Our phylogeny indicates that passenger pigeons and band-tailed pigeons share a common ancestor around 12.4 Mya (95 % CI 9.0–16.3 Mya). This common ancestor diverged from other Old World pigeons during the transition between the Oligocene and the Miocene, around 16.2 Mya (95 % CI 11.7–20.5 Mya). This result contrasts with the results of Pereira et al. [5], which placed the split of *Patagioenas*/*Ectopistes* and the remaining Columbids around 35 Mya.

Rapid diversification, such as that identified here for the Columbiformes, may lead to variation among gene trees due to the effects of incomplete lineage sorting [32, 33], which can lead to inference of different phylogenies for different loci. In future, therefore, it will be important to confirm the evolutionary hypotheses presented here using multiple, independently inherited markers. The use of additional calibration points will also likely increase the precision of the nodes age estimates. Nonetheless, the strong support for the branching order of our phylogeny provides new insights into many of the cryptic evolutionary relationships among pigeons and doves, and attests to the resolving power of complete mitochondrial genomes, at least for inference of the evolutionary history of this locus. Broader taxonomic sampling and the addition of a greater diversity of extinct lineages and calibration points may further resolve the timing and nature of evolutionary diversification within this very diverse group of birds.

## Conclusions

We present a new phylogeny of the pigeons and doves (Columbiformes) based on complete mitochondrial genomes from 24 pigeon species including several extinct or extremely rare species. The branching order in the phylogenetic tree is strongly statistically supported. By including a molecular rate calibration from recent genome-scale analyses, we infer that the lineage of pigeons and doves began to diversify during the Oligocene/Miocene transition, which is a more recent diversification than has been suggested previously. We interpret the phylogenetic results in the context of previous research, and support the recognition of the genus *Alopecoenas*.

## Methods

### DNA extraction and sequencing

We obtained recent or historic tissues for 16 pigeon and dove specimens for the purposes of generating mitochondrial genomes. This included bone powder for the dodo and solitaire, feather for the spotted green pigeon and tooth-billed pigeon, and toe pads for all other samples (Table 1). For modern samples we used muscle tissue for mourning dove, muscle and blood tissue for band-tailed pigeon, and liver tissue for the Nicobar pigeon.

**Table 1:** Sample information

Species	Museum accession	GenBank Accession	Reference	Common Name	Average mtDNA Coverage
*Alopecoenas salamonis* ^b^ ^c^	AMNH 224546	KX902250		Thick-billed ground dove	205.0
*Caloenas nicobarica* ^b^	N2009-0024	KX902248		Nicobar pigeon	48.6
*Caloenas maculata* ^b^ ^c^	D3538	KX902249	[16]	Spotted green pigeon	977.6
*Chalcophaps indica*	JEM68	HM746789	[19]	Common emerald dove	–
*Columba janthina*	–	KM926619	[60]	Japanese wood pigeon	–
*Columba jouyi* ^b^ ^c^	AMNH 612456	KX902247		Ryukyu pigeon	74.3
*Columba livia*	–	KJ722068	[61]	Rock dove	–
*Columba livia*	–	NC_013978	[42]	Rock dove	–
*Columba rupestris* ^a^	SRS346866	KX902246		Hill pigeon	237.5
*Didunculus strigirostris* ^b^	OMNH 1764	KX902245		Tooth-billed pigeon	22.5
*Ectopistes migratorius* ^b^ ^c^	FMNH 47395	KX902243		Passenger pigeon	239.6
*Ectopistes migratorius* ^b^ ^c^	FMNH 47397	KX902244		Passenger pigeon	238.7
*Gallicolumba luzonica*	JEM 65	HM746790	[19]	Luzon bleeding-heart	–
*Gallus gallus*	–	NC_007236	[62]	Chicken	–
*Geopelia striata*	JEM 72	HM746791	[19]	Zebra dove	–
*Geotrygon violacea*	IC 1166	NC_015207	[19]	Violaceous quail-dove	–
*Goura cristata* ^b^	OMNH 1762	KX902242		Western crowned pigeon	45.0
*Hemiphaga novaeseelandiae*	–	NC_013244	[17]	New Zealand pigeon	–
*Leptotila verreauxi*	ML 795	NC_015190	[19]	White-tipped dove	–
*Otidiphaps nobilis* ^b^	OUMNH 1759	KX902241		Pheasant pigeon	18.5
*Patagioenas fasciata* ^b^	AMNH DOT 14025	KX902239		Band-tailed pigeon	173.5
*Patagioenas fasciata* ^b^	BTP2013	KX902240		Band-tailed pigeon	17.4
*Pezophaps solitaria* ^b^ ^c^	S1B1	KX902238		Rodrigues solitaire	11.1
*Pterocles gutturalis* ^a^	SRP029347	KX902237		Yellow-throated sandgrouse	49.2
*Pterocles namaqua* ^d^	AJB1452	–	[45]	Namaqua sandgrouse	–
*Raphus cucullatus* ^b^ ^c^	ZMUC AVES-105485	KX902236		Dodo	21.4
*Streptopelia chinensis*	–	KP273832	[63]	Spotted dove	–
*Turtur tympanistria*	JEM 60	HM746793	[19]	Tambourine dove	–
*Zenaida auriculata*	ML 840	NC_015203	[19]	Eared dove	–
*Zenaida macroura* ^b^	Zm1	KX902235		Mourning dove	112.8

The table lists the species that were used in the phylogenetic reconstruction. The symbol ^a^indicates a new mitochondrial genome assembly, ^b^means that the specimen was also sequenced as part of the present work. The symbol ^c^denotes an extinct species. The sign ^d^indicates that instead of a complete mitochondrial genome, the following genes were concatenated: 16S, 12S, ATP8, ATP6, CYTB, COIII, COII, COI, ND5, ND4, ND4L, ND3, ND2, ND1.

We processed modern tissue samples at two institutions: the UCSC Paleogenomics Lab Modern Facility (N2009-0024, BTP2013), and the Church Lab, at Harvard University (AMNH DOT 14025, Zm1). For all the recent samples, we extracted DNA using the Qiagen DNeasy Blood & Tissues Kit (Qiagen, USA) according to the manufacturer’s instructions and sheared the resulting DNA into fragments <1,000 base pairs (bp) long. We size-selected DNA from the Nicobar Pigeon and band-tailed pigeon prior to sequencing. Mourning dove DNA was sequenced without size selection.

For historic samples, we extracted DNA and prepared genomic libraries in isolated, purpose-built ancient DNA facilities at four institutions: the UCSC Paleogenomics ab (samples AMNH 612456, OMNH 1764, AMNH 224546, OMNH 1762, AMNH 616460, Zm1, S1B1, OUMNH 1759), the University of Copenhagen (sample ZMUC AVES-105485), the McMaster University Ancient DNA Centre (samples FMNH 47395, FMNH 47396, and FMNH 47397), and Griffith University (sample D3538). At UCSC, we extracted DNA following [11], in which we digested tissues in buffer modified from the Qiagen Blood & Tissue Kit that comprised 150 μL Buffer ATL, 30 μL proteinase K solution, and 20 μL of 1 M dithiothreitol (DTT), in a rotation incubator at 56 °C for 48 h, and then purified DNA using the Qiagen Nucleotide Removal Kit according to the manufacturers protocol. At McMaster University, we extracted DNA using a phenol:chloroform:isoamyl alcohol and chloroform based solution, or “in-house” silica columns with an extraction to binding buffer ratio of 1:2 and 30 μL silica beads [34, 35]. At Copenhagen University, we first drilled 0.01 g of bone powder through the Foramen Magnum from the inside of the braincase of the “Gottorp” Dodo Specimen (ZMUC AVES-105485), not damaging the exterior of the skull, and using appropriate anti-contamination precautions. We then extracted DNA from the bone powder as in [36], and purified the extract following [37]. We then constructed an Illumina sequencing library using a blunt-end protocol [38]. The library was indexed by amplification with Accuprime under the following conditions: 95 °C for 1 min, then 12 cycles of 95 °C for 15 s, 60 °C for 30 s, 68 °C for 30 s, followed by 68 °C for seven minutes. At Griffith University, we extracted DNA from a feather of the single surviving spotted green pigeon specimen following [16]. The resulting DNA was treated with Uracil-DNA Glycosylase (Thermo), the NEBNext End Repair Module (NEB), the NEBNext Quick Ligation Module (NEB) and the Bst DNA Polymerase Large Fragment (NEB), respectively, each time purifying with the MinElute PCR Purification Kit (Qiagen). We prepared sequencing libraries according to [38] and cleaned the resulting libraries with the AxyPrep Mag PCR Clean-Up Kit (Axygen). The libraries were sequenced as 50 bp single-end reads on an Illumina HiSeq 2500 sequencing system at the Macrogen Inc, South Korea. For samples AMNH DOT 14025 and Zm1 DNA extracts were prepared for sequencing using the Illumina TruSeq kit following manufacturer’s protocols.

For samples extracted at McMaster and UCSC, we prepared uniquely-barcoded Illumina sequencing libraries following [38] and cleaned the libraries using Sera-Mag SPRI SpeedBeads (ThermoScientific) in 18 % PEG-8000. We generated paired-end sequence data from pooled libraries using both an Illumina MiSeq (1 × 75 bp) at UCSC and Illumina HiSeq2000 (1 × 100 bp) at the Vincent J. Coates Genomic Sequencing Center at UC Berkeley, the University of Copenhagen, McMaster University and the University of Toronto.

### Assembly of mitochondrial genomes

To assemble a taxonomically diverse data set of pigeons and doves, we downloaded short read files (SRA) from the online database Sequence Read Archive + (http://sra.dnanexus.com) for *Columba rupestris* (accession SRS346866 [39]), *Pterocles gutturalis* (accession SRP029347 [40, 41]) and *Ectopistes migratorius* (accession SRS391366). We assembled the mitochondrial genomes of passenger pigeon specimens FMNH 47395 and FMNH 47397 (SRA accession SRS391366 for both specimens, GenBank accession JQ692598 for FMNH 47397). We assembled a new mitochondrial genome for FMNH 47397 because the previous assembly contains improperly assembled regions (16 s rRNA, ND6, and D-loop).

We processed our sequencing data and the previously published SRAs by removing adapters using SeqPrep (https://github.com/jstjohn/SeqPrep). For the extinct species, sequence fragments were sufficiently short that we also merged the paired reads also using SeqPrep, enforcing a minimum overlap of 10 base-pairs between forward and reverse reads. We mapped the processed reads to the reference mitochondrial genome of *Columba livia* (GenBank accession NC_013978.1 [42]), and other published pigeon mitochondrial genomes using MIA (https://github.com/udo-stenzel/mapping-iterative-assembler), which is an iterative, reference-based, short-fragment assembler designed for circular genomes [43]. For each genome, we required a minimum of three unique molecules (3× coverage) to call a consensus base at each site; otherwise, bases were called as “N.” Finally, we inspected the assemblies by eye using Geneious R8.1 and corrected poorly assembled regions with a second set of iterative mapping assemblies (http://www.geneious.com, [44]).

In addition to the sequences described above, we downloaded previously published mitochondrial data for 12 species of pigeon and dove and chicken (Table 1). We also concatenated mitochondrial genes 16S, 12S, ATP8, ATP6, CYTB, COIII, COII, COI, ND5, ND4, ND4L, ND3, ND2, ND1 from the Namaqua sandgrouse, creating a partial mitochondrial genome (GenBank accessions DQ385063.1, DQ385080.1, DQ385097.1, DQ385114.1, DQ385131.1, DQ385148.1, DQ385165.1, DQ385182.1, DQ385199.1, DQ385216.1, DQ385233.1, DQ385250.1, DQ385267.1, DQ385284 [45]). We aligned all the full mitochondrial genomes and the concatenated sandgrouse mitochondrial genes using MUSCLE [46], and visually checked the alignment using SeaView v.4.5.4 [47].

### Phylogenetic analysis

We partitioned the alignment in order to account for different evolutionary rates along the different regions of the mitochondrial genome. We split the alignment into six distinct partitions: 12S and 16S ribosomal RNA genes, all tRNA genes, the hypervariable region, and three partitions for the protein coding genes, according to their codon position. We used PartitionFinder [48] to select the models of evolutionary evolution for each partition and estimated the phylogenetic relationships among the mitochondrial genomes in our data set using BEAST 1.8.1 [49] and RAxML v.8.2.0 [50]. For BEAST we assumed a lognormal uncorrelated relaxed clock [51] for each partition, and a Birth-Death speciation process [52] for the tree prior. To calibrate the molecular clock, we placed a normal prior on the age of the divergence between Columbiformes and Pterocliformes of 55 Mya ± 15 Mya [2, 3]. We ran six MCMC chains for 30 million states, sampling trees and model parameters every 3000 states. We discarded the first 30 % as burn-in, and visually inspected the remainder for convergence using Tracer v1.6 [53].

Since 3^rd^ codon positions evolve more rapidly than 1^st^ and 2^nd^ codon positions, we tested for evidence of saturation at these sites using the method described in [54]. We found no evidence of saturation (Additional file 1: Figure S3). Nevertheless, since changes in substitution biases can result in systematic error [55], and this is more likely to affect synonymous substitutions, we repeated the BEAST analysis using only 1^st^ and 2^nd^ codon positions of coding genes. We obtained the same topology entirely consistent with the use of the entire alignment, suggesting that our estimation of the topology using the whole alignment is not driven by systematic error.

If rates are too variable over the history of a particular group, dating analyses will be unreliable. The absence of multiple calibration points limits our ability to estimate the extent to which rates may have varied over the history of this clade. However, we found that the standard deviation (SD) in the rate variation over the tree, as obtained by BEAST, is sufficiently small to suggest a good fit of a molecular clock model (8.627E-4 SD). Despite this, the determinants of rate variation might be heritable [56], and may, therefore, be better reflected by an autocorrelated clock model. Therefore, in addition to the uncorrelated relaxed clock method from BEAST, we also ran a dating analysis with an autocorrelated rate model using MCMCTREE [57]. We used the same calibration as used for the BEAST analysis, and the same partitioned dataset. The 95 % HPD (highest posterior density) for estimates of node age obtained from the two Bayesian approaches all overlap and are reported in Additional file 2: Table S1 and Additional file 3: Figure S2.

We performed a maximum likelihood (ML) phylogenetic analysis on the same data set using RAxML v.8.2.0 [50]. We assumed the GTRGAMMA model for each partition, and performed 1000 bootstrap replicates.

To evaluate the robustness of our molecular clock approach, we performed an additional analysis in which we estimated divergence times at the nodes in our phylogeny using Reltime [58]. Reltime uses a maximum likelihood approach to calculate branch lengths in substitutions per site and computes branch-specific relative rates without calibrations. We used the topology generated by BEAST as input tree and a GTR evolutionary model with four gamma categories. We then converted the relative divergence time into absolute times using the same calibration as used in the BEAST analysis, except that Reltime treats the calibration information as a flat time range. All Reltime calculations were done in MEGA7 [59]. The ages estimated using this approach fell within the 95 % HPD of estimates from the BEAST and MCMCTREE analyses (Additional file 1: Table S1, Additional file 2: Figure S2), with slightly older mean results compared to BEAST. Based on these results, we infer that while additional calibration points may increase precision in node age estimates, their inclusion is not likely to alter the shape of the tree topology significantly.

## Additional files

Additional file 1: Figure S3. Time tree obtained using the BEAST method. Each branch is colored according to the gradient on the top left of the figure, from lower to higher rate estimates for the coding genes. Each branch has been labelled with its rate. (PDF 185 kb)
Additional file 2: Table S1. Divergence times estimates from BEAST, MCMCTREE and Reltime, including minimum and maximum 95 % CI. Node numbers refer to Additional file 1: Figure S1. (XLSX 10 kb)
Additional file 3: Figure S2. A) Time tree obtained using the Reltime method. Each node received a number. B) Dated nodes, including 95 % CI. Brown circles denotes BEAST results, yellow squares MCMCTREE results, and blue circles Reltime results. Node ID relates to node numbers in panel A, and can be seen in table format in Additional file 1: Table S1. (PDF 250 kb)

## Abbreviations

12S: 12S ribosomal RNA
16S: 16S ribosomal RNA
ATP6: ATP synthase subunit 6
ATP8: ATP synthase subunit 8
bp: Base pairs
COI: Cytochrome oxidase subunit I
COII: Cytochrome oxidase subunit II
COIII: Cytochrome oxidase subunit III
CYTB: Cytochrome b
MtDNA: Mitochondrial DNA
Mya: Million years ago
ND1 NADH: Ubiquinone oxidoreductase core subunit 1
ND2NADH: Ubiquinone oxidoreductase core subunit 2
ND3 NADH: Ubiquinone oxidoreductase core subunit 3
ND4 NADH: Ubiquinone oxidoreductase core subunit 4
ND4L NADH: Ubiquinone oxidoreductase core subunit 4 L
ND5 NADH: Ubiquinone oxidoreductase core subunit 5
SRA: Sequence read archive
