# Supplementary figures and images for: Complete mitochondrial genomes of living and extinct pigeons revise the timing of the columbiform radiation
Source: BMC Evol Biol. 2016 Oct 26;16:230. doi: 10.1186/s12862-016-0800-3 (PMC5080718; doi:10.1186/s12862-016-0800-3)

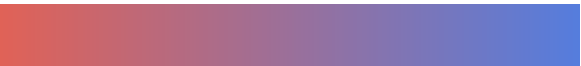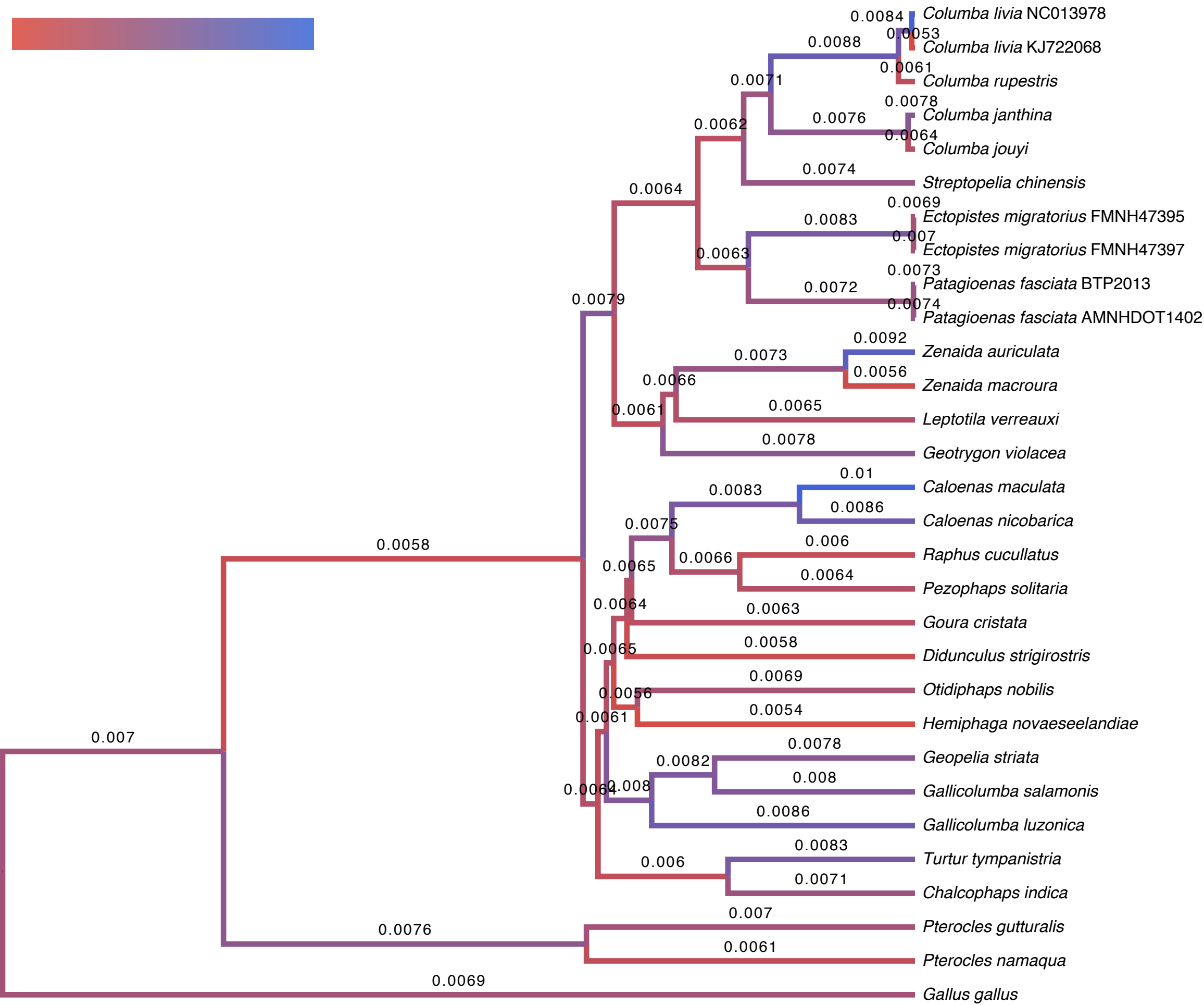

Supplement: Additional file 1: Figure S3. — Time tree obtained using the BEAST method. Each branch is colored according to the gradient on the top left of the figure, from lower to higher rate estimates for the coding genes. Each branch has been labelled with its rate. (PDF 185 kb) [file 12862_2016_800_MOESM1_ESM.pdf]

A

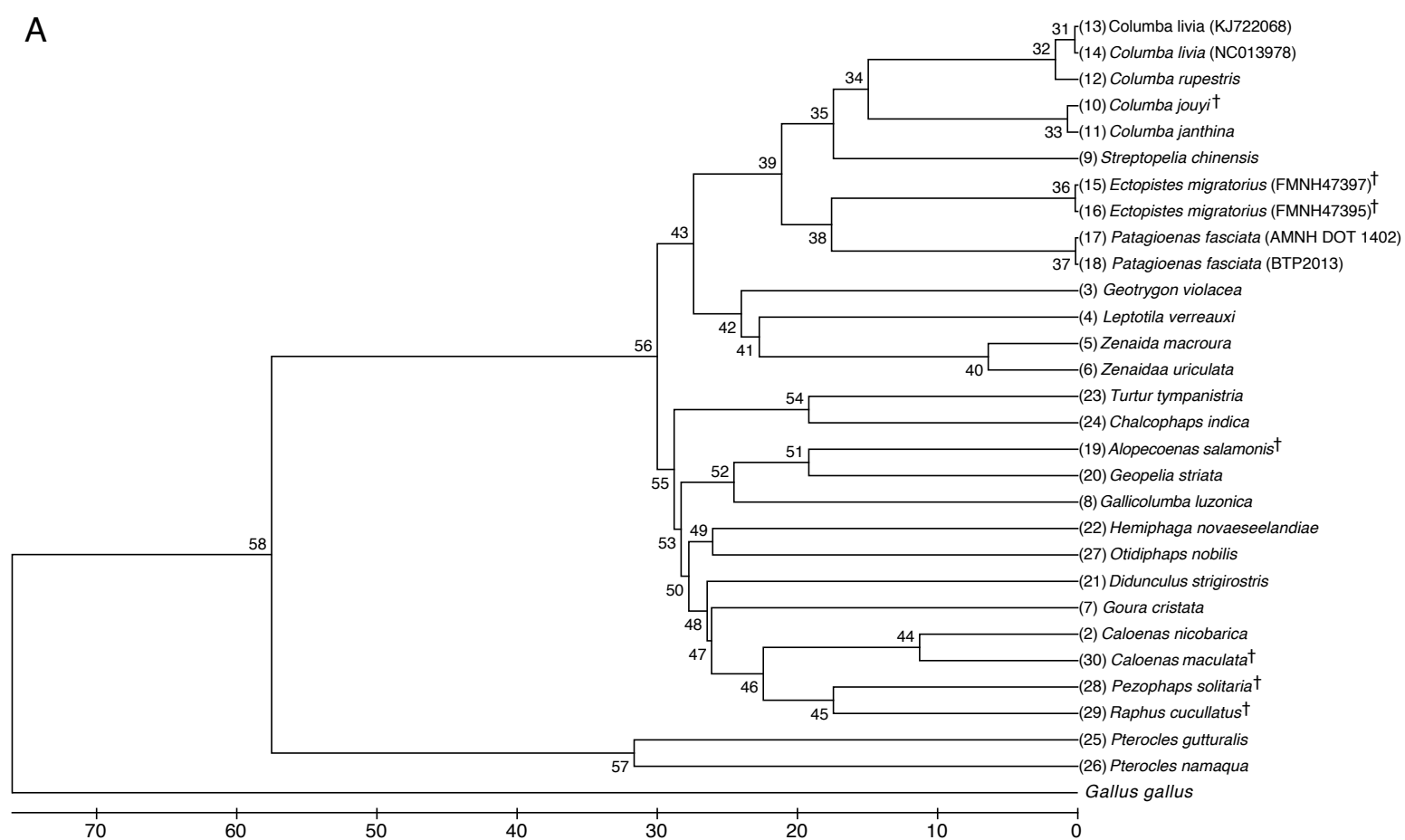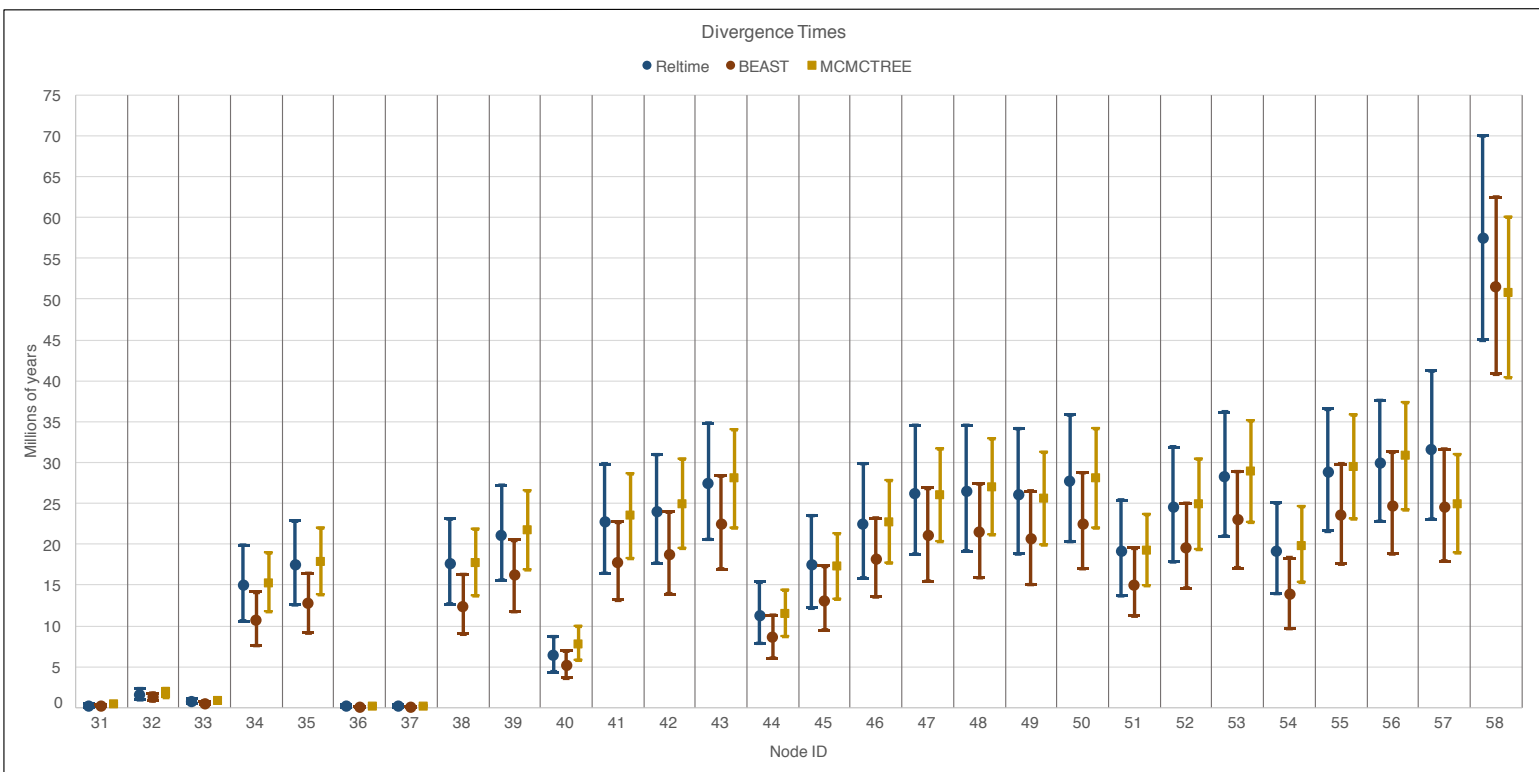

Supplement: Additional file 3: Figure S2. — A) Time tree obtained using the Reltime method. Each node received a number. B) Dated nodes, including 95 % CI. Brown circles denotes BEAST results, yellow squares MCMCTREE results, and blue circles Reltime results. Node ID relates to node numbers in panel A, and can be seen in table format in Additional file 1: Table S1. (PDF 250 kb) [file 12862_2016_800_MOESM3_ESM.pdf]
